# *Brucella* Omp25c Modulates Host NAD^+^/NADH Homeostasis via Interaction with the Mitochondrial Complex I Assembly Factor Ndufaf2

**DOI:** 10.3390/cimb48050472

**Published:** 2026-05-01

**Authors:** Lina Wang, Lian Wu, Kexin Zhang, Rui Ma, Shurong Chen, Tong Ji, Min Zhou, Jiayi Xie, Lingli Zheng, Qingshan Bill Fu

**Affiliations:** 1 School of Chinese Materia Medica, Nanjing University of Chinese Medicine, Nanjing 210023, China; wanglina1056@zidd.ac.cn (L.W.); zhangkexin1057@zidd.ac.cn (K.Z.); marui1058@zidd.ac.cn (R.M.); jitong1731@zidd.ac.cn (T.J.); 2 Zhongshan Institute for Drug Discovery, Shanghai Institute of Materia Medica, Chinese Academy of Sciences, Zhongshan 528400, China; wulian1087@zidd.ac.cn (L.W.); chenshurong@simm.ac.cn (S.C.); zhoumin1845@zidd.ac.cn (M.Z.); xjy22420620@smu.edu.cn (J.X.); zhenglingli@simm.ac.cn (L.Z.); 3 State Key Laboratory of Discovery and Utilization of Functional Components in Traditional Chinese Medicine, School of Pharmaceutical Sciences, Guizhou Medical University, Guiyang 561113, China; 4 University of Chinese Academy of Sciences, Beijing 100049, China; 5 School of Pharmaceutical Sciences, Southern Medical University, Guangzhou 510515, China

**Keywords:** brucellosis, outer membrane protein 25c, NADH dehydrogenase (ubiquinone) complex I assembly factor 2, NAD^+^/NADH

## Abstract

Brucellosis, acting as a typical chronic zoonotic disease, is caused by the invasion of *Brucella* into the human body. Outer membrane protein 25 (Omp25), specifically localized on the *Brucella* membrane, is the main virulence factor of *Brucella* and participates in multiple links of the damage process. Omp25c, a porin protein of *Brucella*, is a paralog of Omp25 with high sequence identity. NADH dehydrogenase [ubiquinone] complex I assembly factor 2 (Ndufaf2) has a key function in cell energy metabolism, particularly in the formation and activity of the mitochondrial respiratory chain. Loss of Ndufaf2 results in oxidative stress and mitochondrial DNA (mtDNA) deletion. However, the functional relationship between Omp25c and Ndufaf2, the underlying mechanism of the proteins, remains unclear. In this work, we purified the Omp25c and Ndufaf2proteins. Our data revealed that Omp25c directly interacts with Ndufaf2, as determined using Biacore analysis. In addition, assays revealed that Ompa2c reshapes the host cell’s redox environment by decreasing the oxidized nicotinamide adenine dinucleotide/reduced nicotinamide adenine dinucleotide (NAD^+^/NADH) ratioand adenosine triphosphate (ATP) production, whereas Ndufaf2 exerts an opposing regulatory effect; Co-expression results further revealed an antagonistic relationship between the two during metabolic processes. These findings provide a new perspective for elucidating the mechanisms of mitochondrial functional regulation in *Brucella*–host interactions and lay the theoretical and experimental foundation for drug development targeting metabolic interventions to eliminate intracellular pathogens.

## 1. Introduction

Brucellosis, a classic chronic zoonotic disease, results from infection with *Brucella* species and can affect both people and animals [1]. To date, brucellosis has spread worldwide and remains endemic in many countries and regions, posing a serious threat to public health and causing substantial losses to the livestock industry. In infected animals, brucellosis commonly leads to reproductive disorders, including miscarriage, infertility, and sterility [2]. In humans, the disease often manifests as a persistent infection, resulting in reproductive system damage and severe clinical manifestations, including meningitis, arthritis, and endocarditis. These outcomes can cause long-term disability and, in severe cases, be life-threatening, thereby imposing considerable economic burden and public health risks [3]. *Brucella* is classified as a facultative intracellular microorganism that is non-spore-forming, non-motile, and lacks flagella [4]. It possesses a capsule and preferentially infects host trophoblasts and macrophages. Despite extensive research, the molecular mechanisms underlying *Brucella* pathogenesis remain incompletely understood [5]. Nevertheless, accumulating evidence indicates that *Brucella* can survive and replicate within host cells. One proposed strategy involves the production of bacterial factors that interfere with phagosome–lysosome fusion, thereby preventing lysosomal antimicrobial components from exerting their functions and enabling *Brucella* to evade host immune clearance [6,7].

In Gram-negative bacteria, outer membrane proteins (OMPs) form essential elements of the outer membrane and are critically involved in mediating interactions with host cells. Because OMPs reside on the bacterial surface, they are readily recognized by the host immune system, triggering antibody production and cellular immune responses [8]. As key determinants of pathogenicity, OMPs contribute to resistance against intracellular killing, maintenance of outer membrane integrity, and adaptation to environmental stresses encountered during infection [9,10]. Among *Brucella* OMPs, outer membrane protein 25 (Omp25) belongs to the group III OMP family and has become a major focus in studies of *Brucella*-induced host damage [11]. Omp25 is considered an important virulence-associated factor and participates in multiple stages of infection. Notably, Omp25 exhibits high conservation among different *Brucella* species, with sequence homology exceeding 98% [12]. Bioinformatic analyses predict a transmembrane region at amino acid positions 6–23, suggesting a role in maintaining outer membrane structural integrity and enhancing bacterial resistance to host killing mechanisms [13]. Consistent with this, Omp25 has been implicated in stabilizing the bacterial outer membrane, which critically contributes to *Brucella* virulence. The integrity of the outer membrane is essential for pathogenicity, as strains with intact outer membranes display increased resistance to professional phagocytes. By reinforcing the outer membrane barrier and modulating host immune recognition, *Brucella* can reduce effective immune activation, thereby facilitating cellular invasion, intracellular survival, and the establishment of both acute and chronic infections [14,15].

Three paralogs have been reported within the Omp25 family, including Omp25b, Omp25c, and Omp25d. Among them, Omp25c is a porin-like outer membrane protein of *Brucella* and shares notable sequence identity with Omp25 and Omp31 [16]. As an important member of the *Brucella* OMP family, Omp25c is implicated in immune regulation and has been proposed as a protective antigen capable of inducing specific antibody and cellular immune responses, positioning it as a promising candidate for vaccine formulation [17]. In addition, Omp25c participates in the regulation of *Brucella* virulence, and loss of Omp25c has been reported to attenuate bacterial virulence and impair intracellular survival within host cells [16]. Beyond its immunological and virulence-associated roles, Omp25c also contributes to outer membrane structural stability and integrity, likely functioning in coordination with other OMPs to maintain the overall architecture of the bacterial envelope [18,19].

Ndufaf2 is essential for cellular energy metabolism, as it supports both the assembly and proper function of the mitochondrial respiratory chain [20]. Ndufaf2 is localized in mitochondria and functions as an assembly factor rather than a structural component of the mature respiratory chain complex. Its primary role is to promote the correct assembly, stability, and maintenance of mitochondrial complex I [21]. A review of the literature reveals that Complex I acts as the ‘gatekeeper’ of the respiratory chain; it catalyzes the first step in NADH oxidation and increases the NAD^+^/NADH ratio, enabling protons to cross the inner mitochondrial membrane, ultimately leading to energy production [22]. Ndufaf2 is an essential chaperone for the normal NADH dehydrogenase function of Complex I. By ensuring the correct folding of Complex I, it indirectly maintains NAD^+^/NADH homeostasis; functional abnormalities may lead to an imbalance in NAD^+^/NADH, thereby contributing to tumor invasion or the development of mitochondrial diseases [23]. Current research on the metabolic regulation of *Brucella* primarily focuses on how *Brucella* influences host NAD(H) metabolism via TIR proteins. Studies have used immunoprecipitation to verify that Omp25c interacts with Ndufaf2, suggesting that this may inhibit the oxidative killing activity of macrophages, thereby creating a more favorable environment for *Brucella* to survive within cells [24]. The interface of the Omp25c–Ndufaf2 interaction may represent a potential therapeutic target for effectively blocking the immunosuppressive effects of *Brucella* and restoring normal cellular function; however, no relevant functional studies have yet been reported.

Therefore, in this study, we expressed and purified recombinant Omp25c and Ndufaf2 proteins and investigated their direct interaction in vitro using surface plasmon resonance (SPR). We further transfected Omp25c and Ndufaf2 expression plasmids into HEK293T cells and evaluated the effects of their expression on cellular NAD^+^/NADH metabolism. Changes in the NAD^+^/NADH ratio were measured to determine whether the Omp25c–Ndufaf2 interaction disrupts intracellular redox homeostasis. Our results demonstrate that Omp25c directly binds to Ndufaf2 and modulates cellular metabolic status by altering the NAD^+^/NADH balance.

## 2. Materials and Methods

### 2.1. Construction of Recombinant Plasmids

The *Omp25c* gene sequence (Uniprot#A0A0E1XAL6) and the *Ndufaf2* gene sequence (Uniprot#Q8N183) were designed and submitted to GenScript for the construction of recombinant plasmids. Both plasmids were generated using pET28a (+) as the vector, with NdeI and XhoI as the restriction enzyme sites, and a 6×His tag fused at the N-terminus. Each construct confers kanamycin resistance.

### 2.2. Expression and Purification of Omp25c

To express Omp25c, the recombinant plasmid was transformed into *E. coli* BL21 (DE3) competent cells (Shanghai Weidi Biotechnology Co., Ltd., Shanghai, China). The target strain was then transferred to LB liquid medium (Beijing Solarbio Science & Technology Co., Ltd., Beijing, China) containing 50 μg/mL kanamycin. When the OD600 value of the bacterial suspension reached 0.6 to 0.8, a sample of the bacterial suspension was collected prior to induction. Afterwards, isopropyl β-D-1-thiogalactopyranoside (IPTG) (Shenzhen Selma Biotechnology Co., Ltd., Shenzhen, China) was added to a final concentration of 0.5 mmol/L.After inducing expression at a temperature of 22 °C and a rotation speed of 220 rpm for 16–18 h, centrifugation was performed to precipitate the cells. The precipitate was suspended in the cold denaturing buffer including 0.5% Triton X-100 (Beijing Pulilai Gene Technology Co., Ltd., Beijing, China), 200 μg/mL lysozyme (Shanghai Yuanye Bio-Technology Co., Ltd., Shanghai, China), 1 mM DL-Dithiothreitol (DTT) (Hangzhou Fude Biological Technology Co., Ltd., Hangzhou, China), and 1 mM phenylmethanesulfonyl fluoride (PMSF) (Beijing Solarbio Science & Technology Co., Ltd., Beijing, China). The cell suspension was disrupted by sonication and centrifuged to retain the supernatant. Add the denaturation solution containing 8 M urea (Shanghai Aladdin Biochemical Technology Co., Ltd., Shanghai, China) to the inclusion bodies in a ratio of 1:10 by volume. Mix thoroughly using a handheld homogenizer (Shanghai Xinnuo Instrument Equipment Co., Ltd., Shanghai, China) until no solid particles remain. Disrupt the cells using an ultrasonic crusher and centrifuge for 30 min to obtain the supernatant. Incubate the supernatant with 2 mL Ni-NTA (Yeasen Biotechnology Co., Ltd., Shanghai, China) resin and transfer slurry to the gravity column. Sequentially, wash the column successively with 10 mM imidazole (Shanghai Macklin Biochemical Co., Ltd., Shanghai, China) and finally elute with 10–500 mM imidazole.

The eluted samples are identified by 12.5% sodium dodecyl sulfate polyacrylamide gel electrophoresis (SDS-PAGE) (Yeasen Biotechnology Co., Ltd., Shanghai, China) to determine the purity of the protein. The protein concentration was estimated using Nano-300 (Hangzhou Allsheng Instruments Co., Ltd., Hangzhou, China).

The protein was diluted to approximately 0.1 mg/mL. The diluted sample was transferred to a dialysis bag (Beijing Wokai Biotechnology Co., Ltd., Beijing, China) and dialyzed into different concentrations of urea rehydration solution at a volume ratio of 1:10 for rehydration. The rehydration was carried out at 4 °C, and the dialysis solution was changed every 12 h or so. Finally, the protein was concentrated using a 5 KD ultrafiltration tube (Merck Millipore, Burlington, MA, USA), and the rehydrated protein was identified using 12.5% SDS-PAGE. After sampling, the samples were freeze-dried and subjected to liquid chromatography–mass spectrometry (LC-MS) [25].

### 2.3. Expression and Purification of Ndufaf2

The Ndufaf2 protein was overexpressed in *E. coli* BL21(DE3) cells cultured in LB medium containing 50 μg/mL kanamycin. When the OD600 value of the bacterial solution reached 0.6 to 0.8, the sample of the pre-induction bacterial solution was collected. Expression was then induced by adding IPTG to a final concentration of 0.15 mM, followed by overnight incubation at 18 °C with shaking at 180 rpm. Cells were harvested by centrifugation, and the pellet was resuspended in a buffer containing 0.5% Triton X-100, 200 μg/mL lysozyme, 1 mM DTT, and 1 mM PMSF. After sonication, the lysate was centrifuged, and the supernatant was collected. This supernatant was transferred to a Ni-NTA column and washed sequentially with buffers containing 10 mM, 20 mM, and 50 mM imidazole, followed by stepwise elution with buffers containing 250 mM and 500 mM imidazole. The eluate was then further purified by size exclusion chromatography. The eluted fractions were analyzed by 12.5% SDS-PAGE to identify the purified target protein. After collection, the samples were freeze-dried and subjected to liquid chromatography–mass spectrometry (LC-MS) [26].

### 2.4. Surface Plasmon Resonance (SPR) Assay

SPR experiments were carried out on a Biacore T200 instrument manufactured by GE Healthcare (Chicago, IL, USA) [26]. The interaction between the Omp25c protein and the Ndufaf2 protein was detected according to the standard operating procedure for surface plasmon resonance provided by Sintofera Company (Hangzhou, China). After diluting the purified and reconstituted Omp25c protein with PBS buffer (Shanghai Jizhi Biochemical Technology Co., Ltd., Shanghai, China), it was further diluted 10 times with 10 mM sodium acetate buffer (Cytiva, Marlborough, MA, USA) of different pH values. The Omp25c protein and NaOH solution (Cytiva, Marlborough, MA, USA) were injected for analysis to determine the optimal pH conditions and the concentration of the protein solution. Mix EDC and NHS (Cytiva, Marlborough, MA, USA) in a 1:1 ratio, activate the CM5 chip. Take 194 μL of 10 mM sodium acetate solution with pH 4.0, add 6 μL of Omp25c protein solution, and make the final concentration of Omp25c protein 100 μg/mL. Couple the Omp25c protein to the activated CM5 chip (Cytiva, Marlborough, MA, USA). After the coupling is completed, seal the CM5 chip with the ethanolamine solution in the amino coupling kit. Load the Ndufaf2 protein onto the CM5 chip, then regenerate it with different pH values of hydrochloric acid glycine solution (Cytiva, Marlborough, MA, USA), and compare the binding ability of the ligands on the chip before and after regeneration to determine the appropriate regeneration conditions.

Dilute the Ndufaf2 protein to 200 nM, 100 nM, 50 nM, 25 nM, 12.5 nM and 6.25 nM with running buffer. Run the multi-cycle kinetic detection program to evaluate the kinetic parameters of the interaction between Omp25c protein and Ndufaf2 protein. We used the Flow path 1 without coupled Omp25c protein as a blank control, and set 100nM as the control group to verify the stability of the chip surface. The running conditions of the kinetic experiment include a reaction temperature of 25 °C, an injection flow rate of 30 μL/min, a contact time of 300 s, and a dissociation time of 300 s; the regeneration solution is selected from the pH 1.5 hydrochloric acid glycine solution after screening, with a flow rate of 30 μL/min, and PBS buffer is used as the blank control to run five cycles. After the reaction, data were analyzed using Biacore T200 Evaluation software version 3.2 (Cytiva, Marlborough, MA, USA). use the double reference subtraction method to eliminate the volume refractive effect and non-specific binding; and apply the 1:1 binding model to determine the kinetic and affinity parameters.

### 2.5. Cell Cultures

The medium was changed every 2–3 days. HEK293T human embryonic kidney cells (ATCC, CRL-11268) were cultured in high-glucose DMEM supplemented with 10% FBS (GeminiBio, West Sacramento, CA, USA) in an incubator at 37 °C and 5% CO_2_.

### 2.6. Plasmid Transfection

HEK293T cells were cultured in DMEM (Guangzhou Bede Biological Technology Co., Ltd., Guangzhou, China) supplemented with 10% fetal bovine serum (Shanghai Yuanye Bio-Technology Co., Ltd., Shanghai, China) at 37 °C with 5% CO_2_ and were seeded into six-well plates (Beyotime Biotechnology, Shanghai, China) at approximately 2.0 × 10^5^ cells per well 24 h prior to transfection, by which time the cells had reached 70–80% confluence. Transfection was performed using Lipo2000 reagent (Thermo Scientific, Waltham, MA, USA). Different transfection groups were set up based on the principle that the total DNA amount per well was constant at 2.0 μg, including the single-transfection group of PcDNA3.1-HA-Omp25c, the single-transfection group of PcDNA-Myc-Ndufaf2, and the co-transfection group of the two plasmids. In the co-transfection group, each plasmid was 1.0 μg; meanwhile, the total DNA amount was made up with the corresponding empty vector in the other groups. The plasmid DNA was first diluted to 250 μL with serum-free medium, and the Lipo2000 reagent was diluted with serum-free medium and then mixed with the DNA solution. To form transfection complexes, the mixture was incubated at room temperature for 15 min. The complexes were then evenly added to the cells for transfection. Six hours after transfection, the medium was replaced with fresh complete medium, and the cells were incubated for an additional 48 h before subsequent experimental analysis. Control groups were included in all experiments.

### 2.7. Western Blotting Analysis

The samples were mixed in proportion with the loading buffer (Beyotime Biotechnology, Shanghai, China). An appropriate protein gel concentration was selected to perform electrophoretic separation of proteins of different molecular weights. The proteins separated on the gel were transferred onto a polyvinylidene fluoride (PVDF) membrane (Merck Millipore, Burlington, MA, USA) under the conditions of 90 V for 2 h. Subsequently, the transfer solution remaining on the PVDF membrane was rinsed with Tris-buffered saline containing Tween 20 (Shanghai Macklin Biochemical Co., Ltd., Shanghai, China) three times, with each rinse lasting 5 min. The membrane was first blocked with 5% skim milk for 2 h, and any residual blocking solution was removed by extensive washing. It was then incubated overnight at 4 °C with primary antibodies against Omp25, β-actin (Proteintech Group, Inc., Wuhan, China; dilution ratio to be added), and Ndufaf2. Following incubation, the membrane was washed with Tris-buffered saline supplemented with 0.1% Tween 20, and then incubated with secondary antibodies at room temperature for 2 h. After removing unbound secondary antibodies by washing, the protein bands on the membrane were detected and visualized.

### 2.8. Determination of NAD^+^ and NADH Content

The concentrations of NAD^+^ and NADH were determined according to the instructions provided with the NAD^+^/NADH assay kit (Beyotime Biotechnology, Shanghai, China). After transfection for 48 h, 1 × 10^6^ cells were washed with PBS. Add 200 μL of pre-cooled NAD^+^/NADH extraction solution to each well by gently pipetting to lyse the cells. Incubate for 10 min, then centrifuge to obtain the supernatant, which is the sample to be tested. Pipette 50–100 μL of the sample into a centrifuge tube, and heat it in a water bath at 60 °C for 30 min to fully degrade NAD^+^. Subsequently, incubate at 37 °C for 10 min in the dark. Add 10 μL of chromogenic solution to each well and mix thoroughly. Place the microplate in the dark at 37 °C for 30 min; the reaction initiates immediately upon addition of the chromogenic solution, gradually producing orange-yellow formazan. The OD450 values were measured using a Spark multimode micro-plate reader (Tecan, Männedorf, Switzerland).

### 2.9. Measurement of Intracellular ATP Levels

The purpose of this experiment is to investigate the effects of two plasmids (PcDNA3.1-HA-Omp25c and PcDNA-Myc-Ndufaf2) on cellular ATP levels by transfecting them into HEK293T cells. First, HEK293T cells were cultured in DMEM medium containing 10% fetal bovine serum at 37 °C and 5% CO_2_. Twenty-four hours prior to transfection, the cells were seeded into a six-well plate at a density of approximately 5 × 10^5^ cells per well. Once the cell density reached the expected level, transfection was performed. Subsequent experimental analyses were conducted 48 h later. A non-transfected control group was included in all experiments.

### 2.10. Statistical Analysis

GraphPad Prism 8.0 (GraphPad, San Diego, CA, USA) was utilized for statistical analyses. Data comparison was performed via one-way ANOVA, and *p* < 0.05 was considered statistically significant. Results are shown as mean ± standard deviation.

## 3. Results

### 3.1. Purification of Omp25c

**Figure 1:**
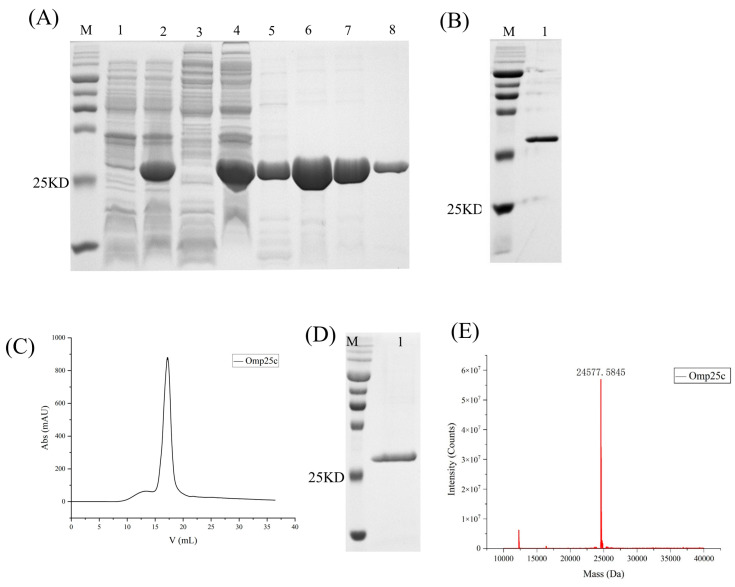
Expression and purification of recombinant Omp25c. (**A**) SDS–PAGE analysis of Omp25c expression and Ni–NTA purification. Lane M, protein marker; lanes 1–2, uninduced and induced *E. coli* BL21(DE3) harboring pET-28a-Omp25c; lanes 3–4, soluble (supernatant) and insoluble (pellet) fractions after cell lysis; lanes 5–8, purified Omp25c eluted with 10, 50, 250, and 500 mM imidazole, respectively. (**B**) SDS–PAGE analysis of Omp25c refolding after stepwise dialysis. Lane M, protein marker; lane 1, refolded Omp25c. (**C**) Size-exclusion chromatography (SEC) profile of purified Omp25c. (**D**) SDS–PAGE analysis of the major SEC peak fraction. Lane M, protein marker; lane 1, purified Omp25c. (**E**) LC–MS spectrum confirming the molecular mass of Omp25c.

The *Omp25c* gene was subcloned into the pET-28a(+) vector, followed by transformation into *E. coli* BL21(DE3). Bacterial cultures collected before and after IPTG induction were analyzed by SDS–PAGE, confirming successful expression of recombinant Omp25c (Figure 1A). The denatured protein was then purified and refolded by stepwise dialysis against decreasing concentrations of urea. Samples collected during the refolding process were examined by SDS–PAGE (Figure 1B). The refolded Omp25c protein was subsequently concentrated and further purified by size-exclusion chromatography (SEC). The SEC elution profile is shown in Figure 1C, and peak fractions were analyzed by 12.5% SDS–PAGE (Figure 1D). Purified samples were lyophilized and subjected to LC–MS analysis (Figure 1E). The measured molecular mass of Omp25c was approximately 24 kDa, consistent with its predicted size, confirming the successful purification of recombinant Omp25c.

### 3.2. Purification of Ndufaf2

**Figure 2:**
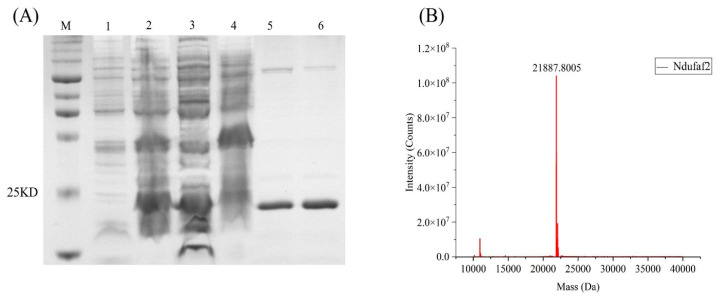
Expression and purification of recombinant Ndufaf2. (**A**) SDS–PAGE analysis of Ndufaf2 expression and purification. Lane M, protein marker; lanes 1–2, uninduced and induced *E. coli* BL21(DE3) harboring pET-28a-Ndufaf2; lanes 3–4, soluble (supernatant) and insoluble (pellet) fractions after cell lysis; lanes 5–6, purified Ndufaf2 eluted with 250 and 500 mM imidazole, respectively. (**B**) LC–MS spectrum confirming the molecular mass of purified Ndufaf2.

After transformation of the recombinant plasmid into *Escherichia coli* BL21(DE3), bacterial cultures collected before and after induction were analyzed by 12.5% SDS–PAGE, confirming the expression of recombinant Ndufaf2 (Figure 2A). The purified Ndufaf2 protein was then lyophilized and subjected to LC–MS analysis (Figure 2B). The major peak corresponded to a molecular mass of approximately 20 kDa, which is consistent with the predicted size of Ndufaf2. These results confirm that recombinant Ndufaf2 was successfully purified.

### 3.3. Kinetic Parameters of the Interaction Between Omp25c and Ndufaf2

Surface plasmon resonance (SPR) was performed to characterize the interaction between Omp25c and Ndufaf2 and to determine the corresponding kinetic parameters. In this assay, Omp25c (24 kDa) was immobilized on a CM5 sensor chip as the ligand, while Ndufaf2 (20 kDa) was injected as the analyte. According to the supplier’s Expasy SOP, when the molecular weight ratio between the ligand and analyte is within the range of 1–5, a target immobilization level of approximately 1000 RU is recommended. Therefore, Omp25c was coupled to the CM5 chip until an immobilization response of ~1000 RU was achieved.

**Figure 3:**
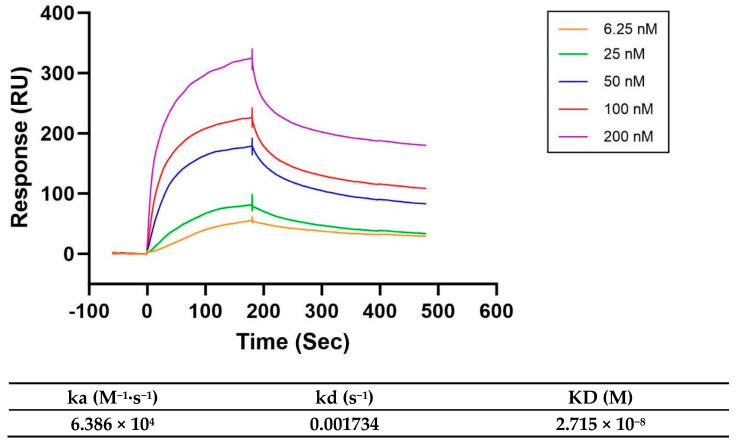
Surface plasmon resonance(SPR) sensorgrams and kinetic fitting of the Omp25c-Ndufaf2 interaction.

The sensorgrams and kinetic fitting results are shown in Figure 3. Analysis of the binding curves yielded an association rate constant (ka) of 6.386 × 10^4^ M^−1^·s^−1^, a dissociation rate constant (kd) of 1.734 × 10^−3^ s^−1^, and an equilibrium dissociation constant (KD) of 2.715 × 10^−8^ M, indicating a high-affinity interaction between Omp25c and Ndufaf2.

### 3.4. Transfection of Omp25c and Ndufaf2 Alters the NAD^+^/NADH Ratio in HEK293T Cells

**Figure 4:**
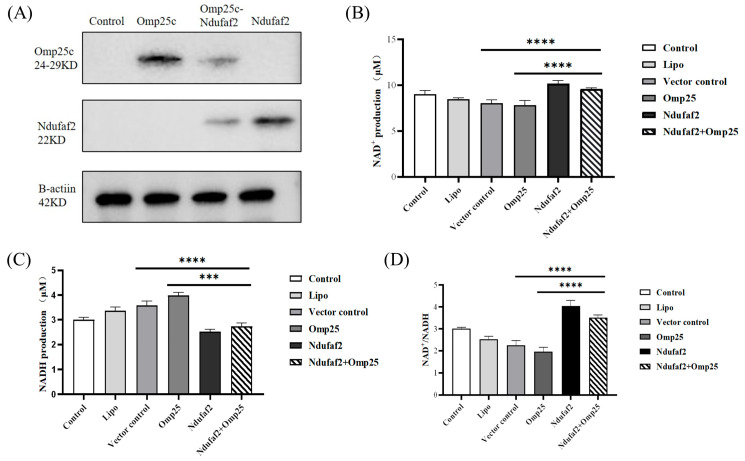
Effects of Omp25c and Ndufaf2 expression on NAD^+^/NADH metabolism in HEK293T cells. (**A**) Western blot verification of plasmid transfection and protein expression. Quantification of (**B**) NAD^+^ levels, (**C**) NADH levels, and (**D**) the NAD^+^/NADH ratio in HEK293T cells transfected with Omp25c and/or Ndufaf2. Data are presented as mean ± SD. Statistical analysis was performed using one-way ANOVA followed by Bonferroni’s multiple comparisons test. *** *p* < 0.001; **** *p* < 0.0001.

Following plasmid transfection, protein expression in each group was verified by Western blotting. The results confirmed successful transfection and expression of the corresponding constructs (Figure 4A). The NAD^+^/NADH ratio is a crucial indicator of cellular redox status and mitochondrial respiratory function. In this study, different transfection conditions produced distinct effects on NAD metabolism in HEK293T cells, and these changes were closely associated with the functional properties of the expressed proteins. Quantification of NAD^+^, NADH, and the NAD^+^/NADH ratio is shown in Figure 4B–D.

Compared with the control group, the Lipo group exhibited a slight decrease in NAD^+^ levels accompanied by a mild increase in NADH, suggesting that the transfection reagent itself may exert a modest influence on cellular redox metabolism. In the empty-vector (Control) group, NAD^+^ levels decreased further relative to the Lipo group, while NADH levels continued to increase, resulting in a marked reduction in the NAD^+^/NADH ratio. These results indicate that the introduction of exogenous DNA may increase cellular metabolic burden and shift redox balance toward a more reduced state.

Among the functional plasmid groups, the expression of Omp25c alone further reduced NAD^+^ levels compared to the empty vector control group, accompanied by a relative increase in NADH, resulting in the lowest NAD^+^/NADH ratio among all groups. This suggests that Omp25c expression may exacerbate the metabolic burden and reductive stress on the cells. In contrast, Ndufaf2 overexpression significantly increased intracellular NAD^+^ levels and decreased NADH levels, resulting in a NAD^+^/NADH ratio that was markedly higher than that of the control group and all other treatment groups, indicating a shift in the cellular redox state toward a more oxidized state.

Compared with the control group and the group transfected with Omp25c alone, NAD^+^ levels were slightly elevated in the group co-transfected with Omp25c and Ndufaf2, while NADH levels were reduced. The NAD^+^/NADH ratio was significantly higher than that in the Control, Lipo, and Vector control groups, but slightly lower than that in the group transfected with Ndufaf2 alone. This suggests that the combined expression of the two proteins exerts a partially additive but not fully synergistic effect on NAD metabolism.

### 3.5. Determination of ATP Content

**Figure 5:**
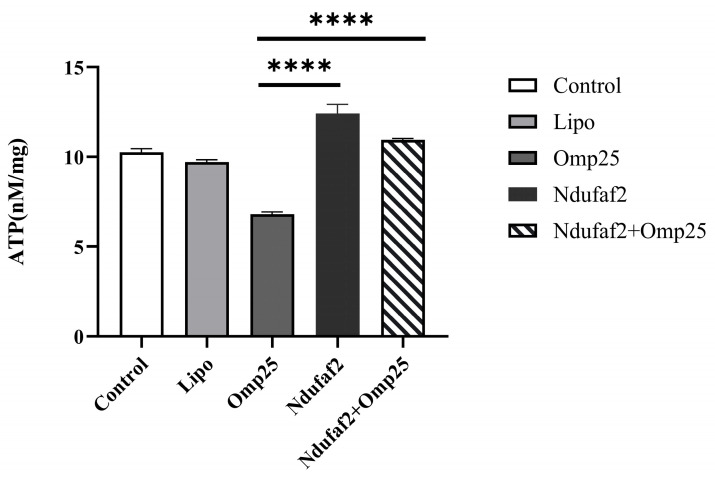
The Effect of Omp25c and Ndufaf2 Protein Expression on ATP Levels. **** *p* < 0.0001.

ATP levels in HEK293T cells were measured using the luciferin–luciferase bioluminescence assay and normalized to total protein content (nmol/mg protein). The results of the ATP content measurement, as shown in Figure 5, indicate that different transfection treatments had a significant impact on cellular energy metabolism. Compared with the Control group, ATP content in the Omp25c-transfected group was significantly reduced, suggesting that its expression markedly inhibits cellular energy production. Conversely, ATP content in the Ndufaf2-transfected group was significantly higher than that in the Control group, indicating an enhancing effect on cellular energy metabolism. In the Omp25c and Ndufaf2 co-transfection group, ATP levels were markedly higher than those in the Omp25c single-transfection group but remained significantly lower than those in the Ndufaf2 single-transfection group. At the same time, ATP levels in this group were still higher than those in the Control group, indicating that the promoting effect of Ndufaf2 on ATP production persists under co-transfection conditions, although it is partially attenuated by the co-expression of Omp25c.

## 4. Discussion

Previous studies have shown that a *Brucella* suis mutant strain lacking the *Omp25c* gene fails to cause disease in the host, despite retaining the ability to proliferate intracellularly. Compared with the parental strain, the Δomp25 mutant exhibits attenuated virulence and a reduced capacity to induce humoral immune responses, indicating that Omp25 functions as both a virulence factor and an immunogenic protein [27,28]. A review of the literature revealed that Omp25c belongs to the *Brucella* Omp25 protein family and shares a high degree of homology with Omp25 [29]. A recent study has confirmed that when Omp25 is expressed alone in HEK293T cells, it localizes to the endoplasmic reticulum (ER), and this process is independent of the bacterial membrane environment. Similar to Omp25, Omp25c contains a conserved N-terminal signal peptide, which enables it to be recognized by the eukaryotic secretory system; therefore, it is reasonable to infer that Omp25c would localize to the endoplasmic reticulum (ER) in mammalian cells [30].

Ndufaf2 was initially identified as a Myc-controlled gene associated with the proliferation of esophageal carcinoma cells [31], and its transcript and protein expression have been reported to be regulated by the inflammatory cytokine IL-1 [32]. In addition, as a mitochondrial protein, the functional interaction between Ndufaf2 and the endoplasmic reticulum-localized Omp25c may depend on specialized membrane contact sites between the endoplasmic reticulum and mitochondria—mitochondria-associated membranes (MAMs). MAMs maintain the spatial proximity of the two organelles, providing a critical structural foundation for their direct interaction. Previous studies have validated the interaction between Omp25c and Ndufaf2 using yeast two-hybrid and immunoprecipitation assays. These experimental results align with the structural support provided by MAMs, further substantiating the validity of this experimental system. Building on this, surface plasmon resonance (SPR) technology can address the limitations of yeast two-hybrid and immunoprecipitation experiments. By measuring the equilibrium dissociation constant (KD) between the two proteins, SPR directly confirms the existence of a high-affinity physical interaction, thereby further validating the authenticity of the MAM-mediated protein interaction and providing a reliable experimental basis for subsequent studies on the mechanisms by which these two proteins regulate cellular metabolism.

The NAD^+^/NADH redox pair is a crucial electron carrier in cellular metabolism. NAD^+^ is the oxidized form, while NADH is the reduced form. During catabolic processes (such as glycolysis and the tricarboxylic acid cycle), NAD^+^ acts as an electron acceptor, accepting the electrons and hydrogen released from substrates and being reduced to NADH; subsequently, NADH transfers electrons to the mitochondrial electron transport chain and is oxidized back to NAD^+^. This reversible redox cycle maintains the energy homeostasis and redox balance within the cell. Complex I serves as a key hub in regulating the NAD^+^/NADH metabolic balance, and the Ndufaf2 protein, as a crucial factor in the assembly of Complex I, plays an irreplaceable role in maintaining cellular energy metabolism and redox homeostasis. Although Ndufaf2 is closely associated with the regulation of NAD(H) metabolism, no studies have yet reported whether the *Brucella* outer membrane protein Omp25c binds to host Ndufaf2 to interfere with Complex I function, disrupt the NAD^+^/NADH balance, and disturb cellular metabolic homeostasis.

In this study, recombinant *Brucella* Omp25c and human Ndufaf2 proteins were expressed and purified using a prokaryotic expression system (*Escherichia coli*). Protein quality and purity were further confirmed by size-exclusion chromatography (SEC) and LC–MS analysis. In previous work by Qu Hailong et al., the interaction between Omp25c and Ndufaf2 was supported by yeast two-hybrid screening and co-immunoprecipitation assays. Yeast two-hybrid analysis is suitable for high-throughput identification of potential interacting partners; however, it is associated with relatively high false-positive and false-negative rates and is limited in detecting interactions influenced by post-translational modifications. Co-immunoprecipitation provides higher physiological relevance and enables the investigation of protein interactions in a cellular context, but it cannot distinguish between direct and indirect associations [33]. In addition, this method depends strongly on antibody specificity and may have limited sensitivity for weak or transient interactions [34]. Surface plasmon resonance (SPR) is an in vitro, label-free, real-time, and quantitative technique that allows for continuous monitoring of association and dissociation processes and provides accurate kinetic and affinity parameters without the need for protein labeling [35,36]. Compared with traditional affinity-based assays, SPR offers greater objectivity by capturing the dynamic binding process in real time and enables direct measurement of kinetic constants and binding affinity [37,38]. In the present study, SPR analysis yielded an equilibrium dissociation constant (KD) of 2.715 × 10^−8^ M, indicating a high-affinity interaction and providing direct evidence that Omp25c physically binds to Ndufaf2.

As a key indicator of intracellular redox status, the NAD^+^/NADH ratio reflects the efficiency of cellular energy metabolism and overall cellular health [39]. NAD^+^ and NADH constitute a fundamental redox coenzyme pair that plays an essential role in energy production and diverse cellular functions [40]. NAD^+^ represents the oxidized form, whereas NADH is the reduced form. Together, they form the NAD(H) redox couple, which regulates numerous biochemical reactions through reversible electron and hydrogen transfer [41]. The primary roles of NAD^+^ and NADH are closely linked to energy metabolism and cellular homeostasis. During cellular respiration, NAD^+^ accepts electrons and hydrogen equivalents generated during glycolysis and the tricarboxylic acid (TCA) cycle, thereby producing NADH. NADH subsequently donates electrons to the mitochondrial electron transport chain to support oxidative phosphorylation and ATP generation. In addition to its metabolic role, NAD(H) homeostasis contributes to cellular defense against oxidative stress and is important for maintaining mitochondrial integrity and normal cellular function [42]. In both clinical and research settings, intracellular NAD^+^ levels and the NAD^+^/NADH balance have been associated with aging and multiple disease states. As an indicator of mitochondrial function and metabolic fitness, the NAD^+^/NADH ratio is increasingly used as a biomarker for evaluating cellular health. Emerging studies are also exploring therapeutic strategies targeting NAD^+^ metabolism in metabolic disorders, neurodegenerative diseases, and interventions aimed at delaying aging-related functional decline [43,44].

The decreases in NAD^+^ levels and concomitant increases in NADH observed in the Lipo groups suggest that transfection reagents can impose a measurable metabolic burden and induce mild redox stress. Such effects are commonly detected when using metabolically sensitive readouts, highlighting the importance of including multiple appropriate control groups in NAD-related studies.

Notably, Omp25c expression alone further reduced the NAD^+^/NADH ratio, indicating that Omp25c may disrupt cellular energy metabolism or promote the accumulation of reducing equivalents. This phenotype may be associated with altered mitochondrial function, increased protein homeostasis burden, or activation of cellular stress responses; however, the precise mechanism requires further investigation. In contrast, Ndufaf2 overexpression markedly increased the NAD^+^/NADH ratio. Further analysis indicates that the interaction between Omp25c and Ndufaf2 has significant biological implications. As an assembly cofactor for mitochondrial respiratory chain complex I, Ndufaf2 plays a role in regulating the cellular NAD^+^/NADH redox balance and energy metabolism. By binding to Ndufaf2, Omp25c disrupts the host’s metabolic functions; this interaction provides a key molecular mechanism by which *Brucella* regulates the host’s metabolic environment. During infection, this interaction confers a significant survival advantage to *Brucella*: By inhibiting Ndufaf2 function, Omp25c reduces the host cell’s NAD^+^/NADH ratio and ATP levels, thereby weakening the host’s energy supply and immune defense strength. Simultaneously, it induces a mild, controllable intermediate metabolic phenotype that prevents excessive host cell death while facilitating *Brucella*’s evasion of host clearance and enabling persistent intracellular survival. This regulatory mechanism, based on redox balance and metabolism, is a key strategy for *Brucella* to establish persistent intracellular infection, highlighting the significant value of these findings for understanding pathogen–host interactions. At the same time, in the co-transfection group, the Ndufaf2-mediated increase in the NAD^+^/NADH ratio was still present but was partially attenuated compared with Ndufaf2 transfection alone. This suggests that Omp25c may counteract or limit the metabolic benefits conferred by Ndufaf2, resulting in an intermediate redox phenotype. Importantly, these results indicate that metabolic outcomes during multi-gene co-expression are not simply additive, but may reflect coordinated or antagonistic functional interactions between proteins. In this study, we employed a simultaneous co-transfection protocol to establish an antagonistic relationship between Omp25c and Ndufaf2. Sequential transfection experiments will further help us investigate whether the temporal expression of these two proteins affects cellular phenotypes and whether Omp25c can reverse or modulate the functional effects of Ndufaf2 already present within the cells. This experimental model closely aligns with the natural temporal characteristics of *Brucella* infection. We will conduct relevant studies in subsequent research to further investigate and refine the molecular mechanisms underlying the temporal regulation of these proteins. Collectively, the reduction in NADH and relative increase in NAD^+^ in the Ndufaf2-expressing groups supports improved electron transfer efficiency and a shift toward a more oxidized intracellular redox state, which is generally favorable for oxidative metabolism.

Overall, we systematically evaluated the effects of Omp25c and Ndufaf2 expression on the energy metabolism of HEK293T cells by measuring ATP levels and analyzing NAD^+^/NADH metabolism. The results indicate that Omp25c expression significantly reduces cellular ATP levels, accompanied by a decrease in the NAD^+^/NADH ratio, suggesting an inhibitory effect on mitochondrial energy metabolism. Conversely, Ndufaf2 overexpression significantly increases ATP levels, consistent with its effect of significantly elevating the NAD^+^/NADH ratio, indicating that it promotes the efficiency of oxidative phosphorylation by enhancing respiratory chain function. Under co-transfection conditions, the positive regulatory effect of Ndufaf2 on ATP production and redox metabolism persisted; however, this effect was partially attenuated by the co-expression of Omp25c, revealing a nonlinear interaction between the two proteins in the regulation of energy metabolism. These results further support the conclusions drawn from NAD^+^/NADH experiments at the functional level and provide strong evidence for a deeper understanding of the mechanisms by which these proteins regulate mitochondrial function and cellular metabolic homeostasis.

## Data Availability

The original contributions presented in this study are included in the article. Further inquiries can be directed to the corresponding author.
